# Chlorogenic Acid Inhibits Lipid Deposition by Regulating the Enterohepatic FXR-FGF15 Pathway

**DOI:** 10.1155/2022/4919153

**Published:** 2022-02-25

**Authors:** Xiaolin Ye, Jing Li, Zhe Gao, Dongwei Wang, Hongyu Wang, Jie Wu

**Affiliations:** ^1^Department of Gastroenterology, Beijing Children's Hospital, Capital Medical University, National Center for Children's Health, Beijing 100045, China; ^2^Department of Pediatrics, China Medical University Affiliated with Shengjing Hospital, Shenyang, 110004 Liaoning, China

## Abstract

**Aim:**

Chlorogenic acid (CGA) is a natural polyphenolic compound found in human dietary products. Previous studies have confirmed that CGA has many biological activities, such as regulating glucose and lipid metabolism and improving insulin resistance. However, its underlying mechanisms of action remains unclear. Here, we demonstrate the protective effects and molecular mechanisms of action of CGA in reducing weight gain and hyperlipidemia in mice fed with a high-fat diet (HFD).

**Methods and Results:**

C57BL/6 mice were fed with normal chow or HFD; half of the mice in each group received CGA treatment by oral gavage for 16 weeks. CGA treatment was found to significantly inhibit HFD-induced weight gain and hyperlipidemia and increased energy expenditure by promoting the expression of genes involved in thermogenesis and mitochondrial biogenesis. Furthermore, CGA was shown to inhibit the enterohepatic farnesoid X receptor (FXR) fibroblast growth factor 15 (FGF15) pathway and changes serum bile acid (BA) pool, thereby contributing to the increased expression of cholesterol 7 *α*-hydroxylase (CYP7A1).

**Conclusions:**

CGA increases the metabolic elimination of cholesterol by inhibiting the enterohepatic FXR/FGF15 pathway.

## 1. Introduction

Changes in dietary habits and lifestyle have resulted in hyperlipidemia becoming a major public health problem [1]. Hyperlipidemia is characterized by elevated triglyceride (TG) and/or total cholesterol (TC) levels and is an important risk factor for coronary heart disease, bone health, ischemic stroke, and other cardiovascular and cerebrovascular diseases [2, 3].

Bile acids (BAs) have an important role in eliminating cholesterol from the body. They are essential for maintaining cholesterol homeostasis and preventing the accumulation of cholesterol, TG, and toxic metabolites, as well as damage to organs such as the liver [4]. As the body consumes food and drink, BAs are pumped into the small intestine to complete the absorption of lipids. BAs hydrolase produced by gut microbiota dehydrogenate BAs to form secondary BAs, which are reabsorbed through the portal vein system and returned to the liver to complete the hepatoenteric circulation of BAs [5]. The hepatointestinal circulation of BAs is the basis of lipid metabolism in the body. The synthesis of BAs can consume cholesterol in the body and decrease the level of serum cholesterol.

Many studies have shown that metabolic disorders involving BA synthesis and secretion of cholesterol are important factors in the pathogenesis of hypercholesterolemia, type II diabetes, and atherosclerosis [6, 7]. Thus, the identification of novel targets of hyperlipidemia through BA metabolism is crucial for the prevention and treatment of clinical hyperlipidemia and cardiovascular diseases. The farnesoid X receptor (FXR)-fibroblast growth factor 15 (FGF15) pathway is a negative feedback pathway in the hepatoenteric circulation of BAs, and its main role is to inhibit BA synthesis [8]. BA regulates FGF15 gene transcription and promotes the secretion of FGF15 by activating FXR expression in the small intestine. FGF15 enters the liver through the hepatoenteric circulation and suppresses bile acid synthesis by downregulating the expression of the rate-limiting enzyme cholesterol 7*α*-hydroxylase (CYP7A1) in the classic pathway of BA synthesis [9].

Chlorogenic acid (CGA) is a natural polyphenolic compound found in many foods and fruits. In particular, high CGA levels are found in coffee. CGA has a wide range of biological activities, including antitumor effects, as well as lowering blood pressure and blood lipids, and scavenging free radicals [10, 11]. However, it is still unclear whether CGA can regulate BA metabolism. In this study, the effect of CGA on the expression of the gut-liver FXR/FGF15 axis and serum BA profile were investigated to provide further experimental and theoretical basis for the treatment of hyperlipidemia with CGA.

## 2. Methods

### 2.1. Animals

Animal experiments were approved by the Ethics Committee of Shengjing Hospital Affiliated to the China Medical University (approval no. 2020PS034K). Four-week-old C57BL/6 mice were maintained in the Laboratory Animal Center of Shengjing Hospital Affiliated to the China Medical University (specific pathogen-free facility) under a 12 h light/dark cycle. After adaptive feeding for one week, the mice were randomly divided into a normal diet group and high-fat diet (HFD, Beijing HFK Bioscience Co. No. H10060, total energy 21.91 kJ/g, 60% fat, 20% sucrose, 20% protein) group.

Half of the mice in each group were given an oral gavage of CGA (150 mg/kg). The groups were named as follows: normal diet (NFD), normal diet with CGA (NCGA), high-fat diet (HFD), and high-fat diet with CGA (HCGA), n ≥ 6 per group. The HCGA group was administered with the FXR agonist GW4064 (75 mg/kg/d) or FXR antagonist Z-guggulsterone (Z-Gug) (100 mg/kg/d) by oral gavage seven days before being euthanized. The mice were euthanized at 16 weeks of age. Blood, liver, intestine, and adipose tissue were collected and stored at -80oC for subsequent experiments.

### 2.2. Metabolic Activity Measurement

Metabolic activity was determined using the Promethion Animal Monitoring System (Sable Systems International, USA) as previously described [12]. Oxygen consumption (VO2), carbon dioxide production (VCO2), and heat production were measured on six consecutive days and nights, with at least 24 h for adaptation before data recording.

### 2.3. Biochemical Analysis

For biochemical analyses, blood samples were centrifuged at 3000 rpm for 10 min at 4°C. The supernatant was stored at −80°C. An automatic biochemical analyzer Chemray 800 (Redu Life Sciences Co., Ltd., Shenzhen, China) was used to determine the serum concentrations of TC, TG, total bile acid (TBA), low-density lipoprotein (LDL), high-density lipoprotein (HDL), and alanine aminotransferase (ALT).

### 2.4. Real-Time Quantitative PCR

Total RNA was extracted using TRIzol lysis buffer (Invitrogen, Carlsbad, United States), and the concentration of RNA was determined. A PrimeScript RT reagent kit (TaKaRa, Mountain View, CA, USA) was used to reverse transcribe the RNA samples. A SYBR Premix Ex Kit (TaKaRa, Japan) was used with a Bio-RAD iQ5 real-time PCR detection system to perform PCR as previously described [13]. The primers used in this study are listed in Supplemental Table I.

### 2.5. Western Blot Analysis

RIPA and phenylmethane sulfonyl fluoride lysis buffers were used to extract total protein, and the protein concentration was determined using a BCA protein assay kit as described previously [13]. The samples were separated by SDS-PAGE and transferred onto PVDF membranes. The membranes were incubated with primary antibodies (Supplemental Table II) at 4°C overnight, followed by incubation with secondary antibodies for 2 h the next day. Protein bands were visualized using an Enhanced Chemiluminescence Substrate kit (Thermo Fisher Scientific, Rockford, USA), and the results were analyzed with the ImageJ software.

### 2.6. Histological Analysis

Hematoxylin and eosin (H&E) staining, oil red O staining, and immunohistochemistry were carried out as described previously [14]. Briefly, for immunohistochemical analysis, the intestinal tissue and brown adipose tissue (BAT) sections were incubated with antibodies against the mitochondrial uncoupling protein 1 (UCP1) (Wanleibio, China; 1 : 200) at 4°C for 12 h, followed by incubation with a secondary antibody for 1 h. The images were captured by a laser scanning fluorescence microscope (TCS SP5, Leica, Germany) at 200× and 400× magnification.

### 2.7. Bile Acid Analysis

A stock solution of individual bile acid was mixed and prepared in a bile acid-free matrix to obtain a series of bile acid calibrators at concentrations of 30000, 10000, 3000, 1000, 300, 100, 30, or 10 ng/mL. Certain concentrations of GCA-d4, UDCA-d4, CA-d4, GCDCA-d4, LCA-d4, and CDCA-d4 were compounded and mixed as the internal standard (IS). An ultrahigh performance liquid chromatography coupled to tandem mass spectrometry (UHPLC-MS/MS) system (ExionLC™ AD UHPLC-QTRAP 6500+, AB SCIEX Corp., Boston, MA, USA) was used to quantitate bile acids. Separation was performed on an Agela Venusil MP C18 column (2.1 × 100 mm, 2.5 *μ*m), which was maintained at 50°C. The mobile phase, consisting of 0.1% formic acid in water (solvent A) and acetonitrile (solvent B), was delivered at a flow rate of 0.50 mL/min. The mass spectrometer was operated in a negative multiple reaction mode (MRM) as previously reported [15].

### 2.8. Statistical Analysis

Data were analyzed using SPSS 21.0 statistical software. An independent sample *t*-test was used for comparison between the two groups, while three or more groups were compared by ANOVA and Bonferroni's post hoc test. All the bar plots in this study were generated using GraphPad Prism 8.0 (GraphPad Software, San Diego, USA). A *P* value of less than 0.05 indicated that the difference was statistically significant.

## 3. Results

### 3.1. CGA Treatment Inhibits Diet-Induced Obesity and Improves Lipid Metabolism

**Figure 1:**
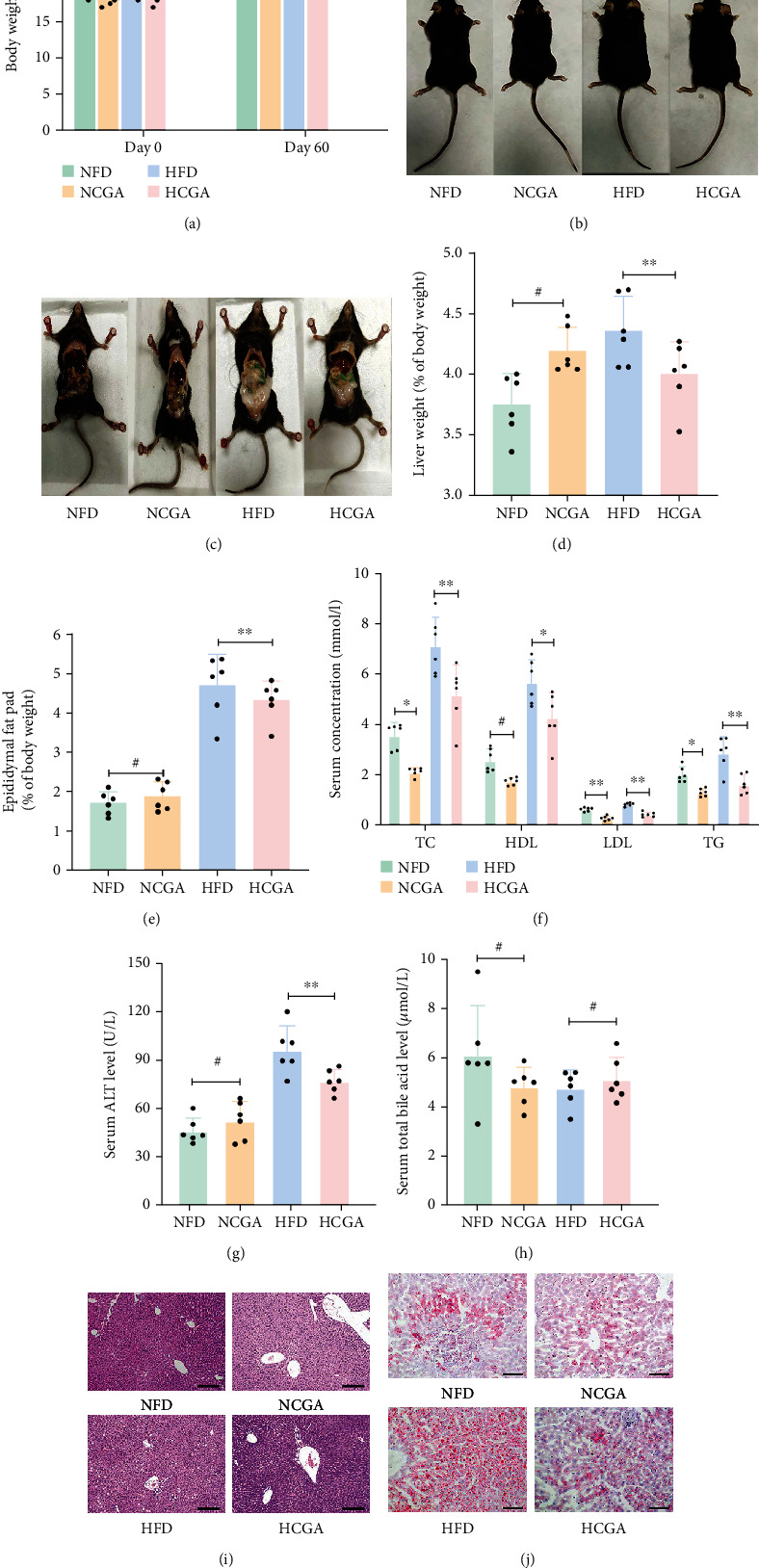
CGA inhibits diet-induced obesity and improves lipid metabolism. (a) Body weights. (b and c) Representative images of mice treated for 16 weeks. (d) Liver weight relative to the total body weight. (e) Epididymal fat pad content relative to the total body weight. (f) Serum TG, HDL, LDL, and TC levels after 6 h fast. (g) Serum ALT levels. (h) Serum TBA levels. (i) Representative images of H&E staining of the liver. (j) Representative images of oil red O staining of the liver. Data are expressed as Mean ± SD. ^#^*P* > 0.05,^∗^*P* < 0.05, and ^∗∗^*P* < 0.01.

Four-week-old male C57BL/6 mice were fed with either normal chow (NFD) or high-fat diet (HFD). Half of the mice in each group received CGA (150 mg/kg) by oral gavage (NFD+CGA, named as NCGA, and HFD+CGA, named as HCGA). After 16 weeks, CGA treatment significantly inhibited the body weight gain of mice in both the NFD and HFD groups (Figure 1(a)). Compared to the NFD group, HFD-fed mice appeared significantly obese and their body fat content had increased significantly. In contrast, the body fat content in HCGA-treated mice was significantly lower than the HFD-fed group (Figures 1(b) and 1(c)). In HFD-fed mice, CGA treatment reduced the percentage of liver and epididymal fat pad weight relative to the total body weight (Figures 1(d) and 1(e)). In addition, CGA intake improved hyperlipidemia as shown by significantly lower TC, LDL, and TG blood levels in the CGA treatment groups (Figure 1(f)). In HFD-fed mice, CGA treatment decreased ALT levels, which is a marker of liver cell damage (Figure 1(g)). No significant differences in TBA levels were observed between the groups (Figure 1(h)). Liver H&E staining revealed that in the NFD group, the liver tissue structure was clear with an orderly arrangement of hepatocytes. Furthermore, no vacuoles were visible in the hepatocytes, and no inflammatory cell infiltration was observed near the portal area. Compared with the NFD group, the structure of the hepatic lobules in the HFD group was disordered, with swollen hepatocytes and seriously damaged cell structure. In addition, an increased amount of lipid droplets and vacuoles was visible in the hepatocytes, and increased inflammatory cell infiltration was observed near the portal area. In contrast, treatment of the HFD group with CGA ameliorated these effects (Figure 1(i)). Oil red O staining revealed an increased amount of lipid droplets in the liver of mice in the HFD group, while CGA treatment was found to inhibit HFD-induced hepatic steatosis (Figure 1(j)).

### 3.2. CGA Enhances Energy Expenditure in BAT

**Figure 2:**
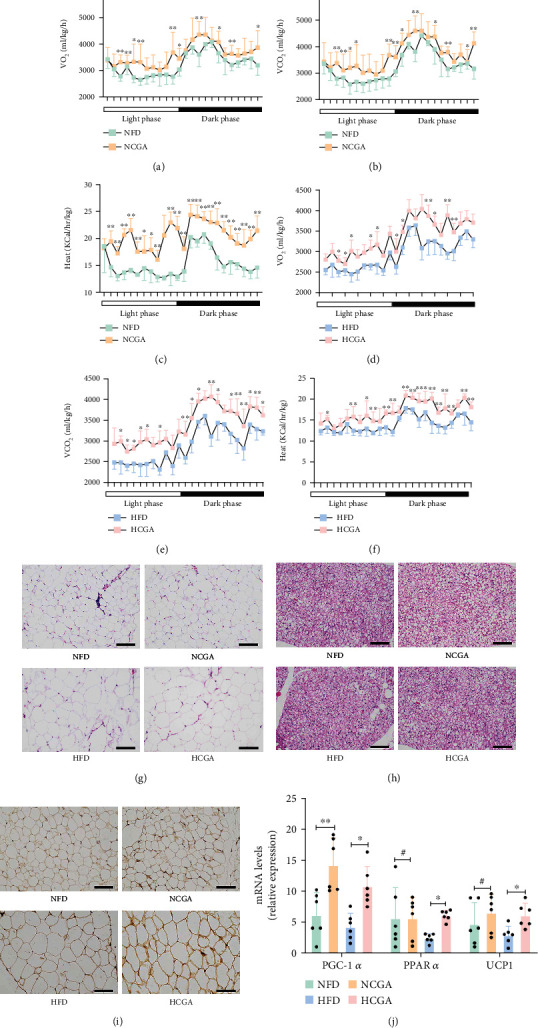
CGA enhances the metabolic rate in BAT. (a and d) Carbon dioxide production. (b and e) Oxygen consumption. (c and f) Heat production. (g) Representative images of H&E staining of epididymal fat. (h) Representative images of H&E staining of BAT. (i) Representative images of UCP1 staining in BAT. (j) Relative mRNA expression of PPAR*α*, PGC-1*α*, and UCP1 in BAT. Data are expressed as mean ± SD. ^#^*P* > 0.05,^∗^*P* < 0.05, and ^∗∗^*P* < 0.01.

We further compared the metabolic rates of mice in each group. We found that in both the HFD- and NFD-fed groups, CGA-treated mice had a consistently higher oxygen consumption (VO2) and exhaled more carbon dioxide (VCO2), as well as increased heat production (Figures 2(a)–2(f)). An increase in the volume and/or number of adipocytes is a pathological sign of obesity. Here, we examined the morphology of the epididymal fat by H&E staining. As shown in Figure 2(g), the epididymal fat volume of HFD mice was significantly larger than that of the NFD mice. Treatment with CGA reduced the volume of adipocytes in the HFD-fed group but had no significant effect on adipocytes in the NFD group. In addition, the prominent accumulation of lipid vesicles in BAT of HFD mice was significantly reduced in CGA-treated mice (Figure 2(h)). Since UCP1 is a major factor involved in the thermogenic process of BAT, we next examined the UCP1 expression in BAT. We found increased UCP1 expression in CGA-treated mice compared to their controls (Figure 2(i)). We also evaluated the effect of CGA on the mRNA expression of peroxisome proliferation-activated receptor alpha (PPAR*α*), the *α*-subunit of peroxisome proliferator-activated receptor-*γ* coactivator-1 (PGC-1*α*) and UCP1, which play an important role in the lipid metabolism, in BAT. Our data indicate that CGA prevents the inhibitory effect of HFD on the expression of adipogenic and thermogenic genes (Figure 2(j)).

### 3.3. CGA Inhibits the Enterohepatic FXR-FGF15 Axis and Changes Serum BA Pool

**Figure 3:**
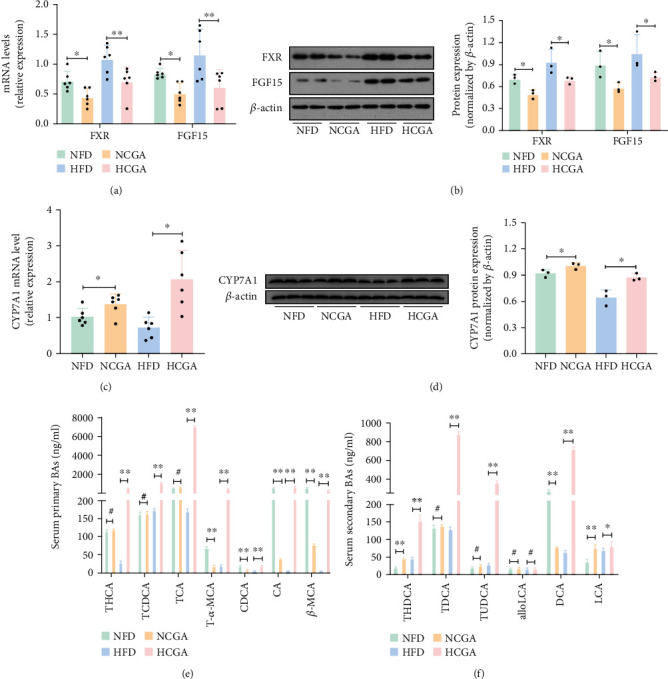
CGA inhibits activation of the gut-liver FXR-FGF15 axis. (a) Relative mRNA expression levels of ileal FXR and FGF15. (b) Relative protein expression levels of ileal FXR and FGF15. (c) Relative mRNA expression levels of liver CYP7A1. (d) Relative protein expression levels of ileal CYP7A1. (e) Serum primary BAs levels. (f) Serum secondary BAs levels. Data are expressed as mean ± SD. ^#^*P* > 0.05,^∗^*P* < 0.05, and ^∗∗^*P* < 0.01.

BAs have been recognized as important signaling molecules to modulate energy metabolism. Previous studies have shown that FXR activation mediates the inhibition of BAs synthesis and induction of FGF15, resulting in the inhibition of the rate-limiting enzyme CYP7A1 [16]. CYP7A1 initiates the classic liver BA synthesis pathway in the liver. Thus, we next sought to determine the role of the gut-liver FXR signal transduction pathway in CGA-induced BA synthesis. We found that CGA treatment led to a significant decrease in ileal FXR and FGF15 mRNA and protein levels (Figures 3(a) and 3(b)). In addition, we found that CGA treatment increased CYP7A1 expression compared with the controls (Figures 3(c) and 3(d)).

BAs are the final product of cholesterol catabolism. The conversion of cholesterol to BAs is a major part of the body's daily cholesterol metabolism. Primary BAs have been shown to protect against obesity and insulin resistance [17]. Thus, we used LC-MS/MS to analyze the composition of the BAs in peripheral circulation. We found that CGA treatment changed the relative proportions of the circulating BAs. In the HFD-fed groups, the primary BAs including THCA, TCDCA, TCA, T-*α*-MCA, CDCA, CA, and *β*-MCA were significantly higher in the CGA-treated mice, with TCA showing the largest increase. A slight, nonsignificant increase in secondary BAs including THDCA, TDCA, TUDCA, DCA, and LCA was also observed in the CGA-treated mice. In the NFD-fed mice, the primary BAs including T-*α*-MCA, CDCA, CA, and *β*-MCA were decreased in the CGA-treated mice, while the secondary BAs THDCA and LCA were increased. DCA was decreased in the CGA-treated mice (Figures 3(e) and 3(f)). These findings indicate that CGA changes serum BA pool; furthermore, altered BA profile may affect the BAT activation by regulating energy homeostasis and thermogenesis.

### 3.4. CGA Inhibits Obesity through Regulation of the FXR-FGF15 Axis

**Figure 4:**
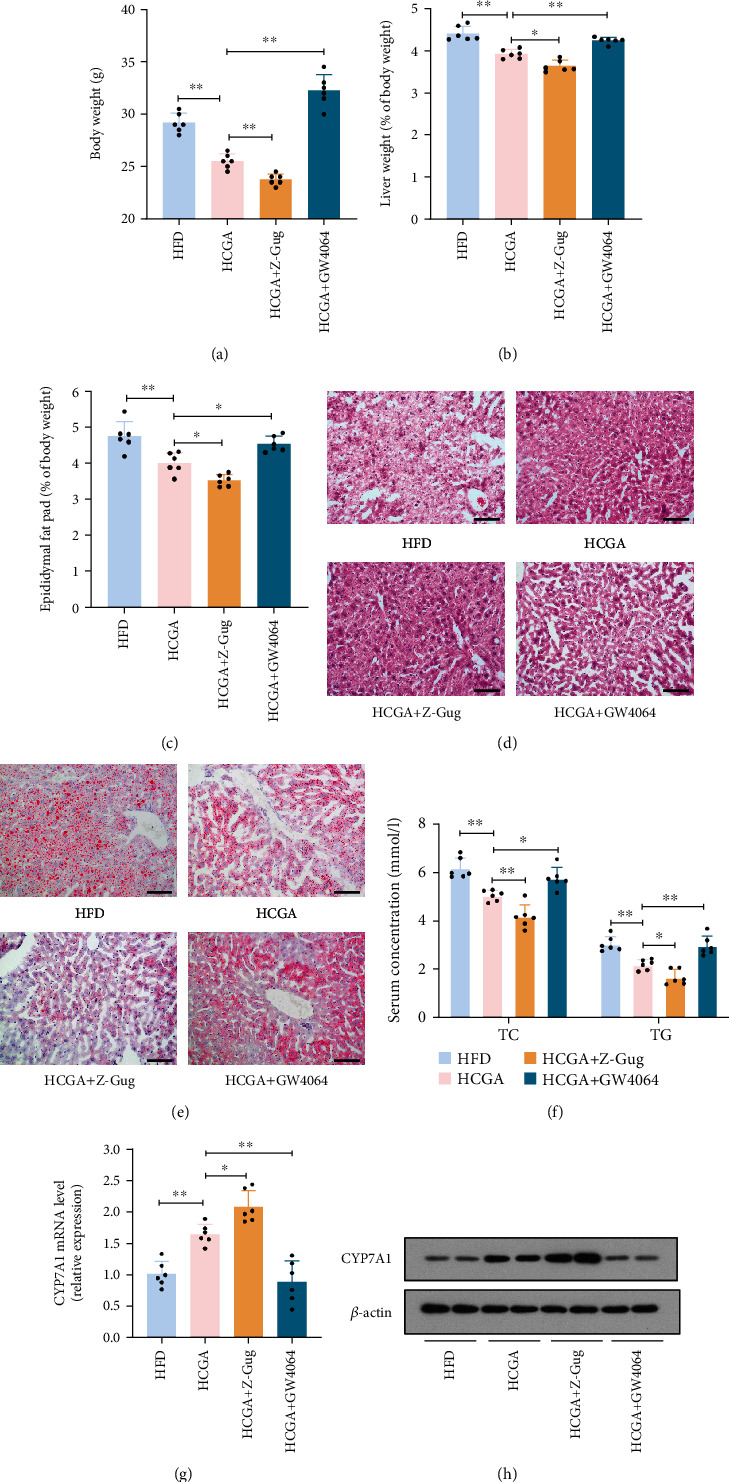
The role of the FXR-FGF15 axis in the improvement of obesity by CGA. (a) Body weights. (b) Liver weight relative to total body weight. (c) Epididymal fat pad content relative to total body weight. (d) Representative images of H&E staining of liver. (e) Representative images of oil red O staining of liver. (f) Serum TG and TC levels after 6 h fast. (g and h) CYP7A1 mRNA and protein expression in liver samples. Data are expressed as mean ± SD. ^#^*P* > 0.05,^∗^*P* < 0.05, and ^∗∗^*P* < 0.01.

To further verify the effect of the enterohepatic FXR-FGF15 axis on the CGA-induced inhibition of obesity, C57BL/6 mice were administered the FXR antagonist Z-Gug and FXR agonist GW4064 to inhibit or increase FXR expression, respectively [18]. Four-week-old male C57BL/6 mice were fed HFD with or without CGA for 16 weeks, and then Z-Gug (100 mg/kg body weight) or GW4064 (75 mg/kg body weight) was given seven days prior to surgical procedures. We found that Z-Gug-treated mice had lower body weights and adipose content compared with the HCGA group, while GW4064 treatment almost reversed the protective effects of CGA against body weight gain (Figures 4(a)–4(c)). Liver H&E and oil red O staining revealed that treatment with Z-Gug inhibited HFD-induced hepatic steatosis, while GW4064 treatment reversed this inhibitory effect of CGA on hepatic lipid accumulation (Figures 4(d) and 4(e)). Serum TG and TC levels were decreased in Z-Gug-treated mice but increased after GW4064 treatment (Figure 4(f)). Furthermore, treatment with Z-Gug markedly increased CYP7A1 expression, while GW4064 treatment almost completely reversed CGA-induced changes in the CYP7A1 expression (Figures 4(g) and 4(h)). In summary, our findings suggest that CGA ameliorates obesity by inhibiting the FXR-FGF15 axis.

## 4. Discussion

Here, we show that CGA has significant metabolic benefits in obese mouse models. CGA protects against diet-induced hyperlipidemia by promoting the expression of genes involved in thermogenesis and mitochondrial biogenesis. Associated with the unexpected browning of white adipose tissue, these changes seem to be mediated in part by a change in BA levels and composition, and this relationship is directly regulated by the gut-liver FXR-FGF15 axis.

Our data demonstrate that CGA treatment markedly increased adipogenic and thermogenic gene expression in BAT. UCP1, mainly found in BAT, participates in thermogenesis regulation and energy metabolism of BAT and maintains the energy metabolism balance of the body [19]. PGC-1*α* is a coactivator of nuclear transcription and plays a role in a series of energy metabolism processes such as adaptive thermogenesis, mitochondrial biosynthesis, liver gluconeogenesis, and fatty acid *β* oxidation [20]. Our findings are consistent with a previous study, which showed that CGA increased PGC-1*α* expression and stimulated mitochondrial oxidative phosphorylation [21]. PPAR*α*, a member of the nuclear receptor superfamily, has an important biological role in inducing downstream target gene transcription. Activation of PPAR*α* can decrease TC levels or hepatic fatty deposition in HFD-fed mice [22]. Huang et al. demonstrated that CGA inhibited obesity through altering the expression of PPAR*α* [23], which is consistent with our findings.

Serum ALT levels are an important indicator of liver injury. Here, we found that CGA significantly reduced ALT levels, which is consistent with previous studies [21]. Liver lipid deposition is not only the basis of the development of nonalcoholic fatty liver disease (NAFLD) but also closely related to obesity, type II diabetes, coronary atherosclerotic heart disease, and other metabolic diseases [24, 25]. Our findings demonstrated that CGA markedly inhibited liver lipid deposition, suggesting that CGA has a protective effect on the liver.

We also demonstrated that CGA significantly altered the level of serum BAs, which are involved in the regulation of glucose and lipid metabolism, energy metabolism, and inflammation in the enterohepatic circulation [26, 27]. BA is important for the removal of cholesterol from the body and is the final product of cholesterol catabolism [28], which have been shown to induce UCP1-dependent thermogenesis and stimulate energy expenditure [29]. BAs are synthesized into primary BAs in the liver through the classic CYP7A1-mediated pathway and the alternative CYP27A1-mediated pathway [30]. CYP7A1 is a rate-limiting enzyme in the classic synthesis pathway, which mediates the synthesis of 75% of all BAs in humans. CYP7A1 catalyzes cholesterol to produce CA and CDCA. The alternative pathway is mediated by CYP27A1 and CYP7B1 and catalyzes the formation of CDCA from cholesterol. Thus, the ratio of CA to CDCA can reflect changes in the classic and alternative pathways of liver BAs synthesis. In this study, CGA markedly increased the CA content, suggesting that CGA may affect the synthesis of BAs by acting on the classic synthesis pathway.

Previous studies have shown that HFD can significantly increase cholesterol and TG levels in the livers of CYP7A1 gene knockout mice [31]. Here, HFD resulted in the downregulation of CYP7A1 in the liver of mice and increased TC, TG, and LDL levels in the serum, similar to the previous reports [32]. After CGA treatment, we observed a significant increase in CYP7A1 gene expression in the liver of HFD mice, while plasma TC, TG, and LDL levels decreased. CYP7A1 can initiate cholesterol catabolism, convert excess cholesterol into BAs, promote BA synthesis, and reduce cholesterol accumulation [33]. Therefore, our current study shows that CGA may increase the rate of BAs synthesis by upregulating the expression of CYP7A1, thereby stimulating the synthesis of BAs, leading to a reduction in blood lipid levels.

Recent studies have shown that the passive intestinal absorption of BAs was increased in HFD-fed mice and led to the activation of the ileal FXR/FGF15 pathway, inhibition of liver CYP7A1 transcription, and reduction in the elimination of cholesterol, finally resulting in liver cholesterol accumulation [34]. The physiological role of FXR as a BA receptor has been demonstrated in FXR knockout mice, with increased BA synthesis being reported in FXR knockout mice [35]. FGF15 is a member of the fibroblast growth factor (FGF) family, and its human homologous gene is FGF19. FGF15 circulates through the liver and inhibits the transcription of CYP7A1 in the classic BAs synthesis pathway [36]. Both CYP7A1 expression and activity and BA synthesis have been shown to be increased in FGF15 knockout mice [37]. In this study, FXR and FGF15 expression levels were upregulated in the ileum of hyperlipidemic HFD-induced mice, while CGA treatment inhibited the activation of the FXR/FGF15 pathway, resulting in increased CYP7A1 expression and BAs synthesis. Many studies have also demonstrated the important role of the FXR/FGF15 axis in the regulation of BAs. For example, the probiotic VSL#3 was found to decrease expression of the FXR/FGF15 axis proteins in the ileum and induce BA synthesis [38]. Similarly, resveratrol significantly reduced FXR mRNA and protein levels, inhibited activation of the FXR/FGF15 axis, increased CYP7A1 mRNA and protein levels, and improved hyperlipidemia [18]. Pu-erh tea also increased the level of ileal conjugated BAs and inhibited the intestinal FXR/FGF15 signaling pathway, resulting in increased production of liver BAs and fecal excretion, decreased liver cholesterol levels, and reduced fat production [39].

## 5. Conclusion

This study provides evidence for the positive effect of CGA on inhibiting HFD-induced weight gain and hyperlipidemia. The current report suggests that daily intake of CGA can upregulate CYP7A1 expression, promote BAs synthesis, and affect BAs metabolism by inhibiting the FXR/FGF15 pathway.

## Data Availability

The data used to support the findings of this study are available from the corresponding author upon request.

## Abbreviations

CGA: Chlorogenic acid
HFD: High-fat diet
FXR: Enterohepatic farnesoid X receptor
FGF15: Fibroblast growth factor 15
CYP7A1: Cholesterol 7*α*-hydroxylase
TG: Triglyceride
TC: Total cholesterol
BAs: Bile acids.

## Supplementary Materials

Supplementary 1 Supplemental Table I: the primers for PCR.

Supplementary 2 Supplemental Table II: primary antibodies for WB.
