# Supplementary material for: Chlorogenic Acid Inhibits Lipid Deposition by Regulating the Enterohepatic FXR-FGF15 Pathway
Source: Biomed Res Int. 2022 Feb 25;2022:4919153. doi: 10.1155/2022/4919153 (PMC8897747; doi:10.1155/2022/4919153)
Supplement: Supplementary 1 — Supplemental Table I: the primers for PCR. [file 4919153.f1.docx]

| Primer | sequence 5' to 3' |
| --- | --- |
| UCP1-F | Sense: GTGAACCCGACAACTTCCGAA |
| UCP1-R | Antisense: TGCCAGGCAAGCTGAAACTC |
| PGC1α-F | Sense:TGAACGCACCTTAAGTGTGGAA |
| PGC1α-R | Antisense: GGGTTATCTTGGTTGGCTTTATGA |
| PPARγ -F | Sense:CACGCATGTGAAGGCTGTAA |
| PPARγ -R | Antisense: GCTCCGATCACACTTGTCG |
| FXR-F | Sense: TGAGAACCCACAGCATTTCG |
| FXR-R | Antisense: GCGTGGTGATGGTTGAATGTC |
| FGF15 -F | Sense: ACGTCCTTGATGGCAATCG |
| FGF15 -R | Antisense: GAGGACCAAAACGAACGAAAT T |
| CYP7A1 -F | Sense:AGCAACTAAACAACCTGCCAGTACTA |
| CYP7A1 -R | Antisense: GTCCGGATATTCAAGGATGCA |
| GAPDH -F | Sense: GCAAAGTGGAGATTGTTGCCAT |
| GAPDH -R | Antisense: CCTTGACTGTGCCGTTGAATTT |

Supplemental Table I. The primers for PCR.

The primers for PCR.
