# Supplementary material for: Chlorogenic Acid Inhibits Lipid Deposition by Regulating the Enterohepatic FXR-FGF15 Pathway
Source: Biomed Res Int. 2022 Feb 25;2022:4919153. doi: 10.1155/2022/4919153 (PMC8897747; doi:10.1155/2022/4919153)
Supplement: Supplementary 2 — Supplemental Table II: primary antibodies for WB. [file 4919153.f2.docx]

| Primary antibody | Dilution ratio | manufacturer | Art.No. |
| --- | --- | --- | --- |
| CYP7A1 antibody | 1：1000 | abcam | ab65596 |
| FGF15 antibody | 1：3000 | abcam | ab225942 |
| FGF15 antibody | 1：3000 | abcam | ab225942 |

Supplemental Table.II. Primary antibodies for WB.
